# Designing Nutritionally Adequate and Climate-Friendly Diets for Omnivorous, Pescatarian, Vegetarian and Vegan Adolescents in Sweden Using Linear Optimization

**DOI:** 10.3390/nu13082507

**Published:** 2021-07-22

**Authors:** Patricia Eustachio Colombo, Liselotte Schäfer Elinder, Anna Karin Lindroos, Alexandr Parlesak

**Affiliations:** 1 Department of Global Public Health, Karolinska Institutet, 113 65 Stockholm, Sweden; liselotte.schafer-elinder@sll.se; 2 Centre for Epidemiology and Social Medicine, Region Stockholm, 113 65 Stockholm, Sweden; 3 The Swedish Food Agency, 751 26 Uppsala, Sweden; annakarin.lindroos@slv.se; 4 Department of Internal Medicine and Clinical Nutrition, The Sahlgrenska Academy, University of Gothenburg, 405 30 Gothenburg, Sweden; 5 Baden-Wuerttemberg Cooperative State University, 74076 Heilbronn, Germany; alexandr.parlesak@heilbronn.dhbw.de; 6 Department of Nutrition, Exercise and Sports, University of Copenhagen, 1165 Copenhagen, Denmark

**Keywords:** planetary health, Paris agreement, linear programming, nutrition, greenhouse gas emission, alternative diets, sustainability

## Abstract

Low-carbon diets can counteract climate change and promote health if they are nutritionally adequate, affordable and culturally acceptable. This study aimed at developing sustainable diets and to compare these with the EAT-Lancet diet. The Swedish national dietary survey Riksmaten Adolescents 2016–2017 was used as the baseline. Diets were optimized using linear programming for four dietary patterns: omnivores, pescatarians, vegetarians and vegans. The deviation from the baseline Riksmaten diet was minimized for all optimized diets while fulfilling nutrient and climate footprint constraints. Constraining the diet-related carbon dioxide equivalents of omnivores to 1.57 kg/day resulted in a diet associated with a reduction of meat, dairy products, and processed foods and an increase in potatoes, pulses, eggs and seafood. Climate-friendly, nutritionally adequate diets for pescatarians, vegetarians and vegans contained fewer foods and included considerable amounts of fortified dairy and meat substitutes. The optimized diets did not align very well with the food-group pattern of the EAT-Lancet diet. These findings suggest how to design future diets that are climate-friendly, nutritionally adequate, affordable, and culturally acceptable for Swedish adolescents with different dietary patterns. The discrepancies with the EAT diet indicate that the cultural dietary context is likely to play an important role in characterizing sustainable diets for specific populations.

## 1. Introduction

All regions around the world are facing severe consequences of global warming [1], resulting in adverse effects on human health and the economy [2]. So far, more than 95% of parties to the United Nations Framework Convention on Climate Change (UNFCCC) have ratified the Paris Agreement, which commits governments to pursue actions to keep the increase in global average temperatures below 1.5 °C above pre-industrial levels and thus prevent dramatic climate change [3]. To reach this goal, environmental, social, and economic aspects of sustainability have to be considered. In the aftermath of the ratification of the Paris Agreement in October 2016 [4], Sweden adopted a climate policy framework [4] with the long-term goal of becoming a net-zero carbon economy by 2045 [4].

Food production contributes globally to about 25–30% of all anthropogenic greenhouse gas emissions (GHGE), through altered land use, storage, transport, packaging, processing, retail, and preparation for consumption [1]. Hence, successful transition into a society that produces less GHGE requires changes at all levels of the food chain. In a market economy, consumer demand is one of the most relevant ways to achieve these changes [5]. Such changes would require a shift towards more plant-based diets, which are generally less GHGE intensive [6,7,8,9,10]. As in other countries [11,12], the motivation to switch to environmentally friendly diets is more pronounced in younger than in older people in Sweden. According to the Swedish Youth Barometer, about a third of all young people are currently consuming more plant-based diets for environmental reasons [13]. However, guidance is needed that can guarantee nutritional adequacy when initiating major dietary changes.

Promoting diets that omit entire food groups such as vegan diets can lead to nutritional deficiencies such as inadequate intakes of calcium, vitamin B12, vitamin D and iron [14], as well as a too-high intake of sugar [14,15]. The choice of foods to replace meat has also been shown to be questionable from a climate perspective as, on a per calorie basis, the substitution of meat products with increased fruit and vegetables can result in higher or similar environmental impacts [6,7,8]. Thus, consumers who want to change their diet to be more climate friendly, yet nutritionally adequate, face a challenge when having to combine foods to meet all these demands. Average dietary intakes of Swedish adolescents are far from meeting the dietary guidelines that aim at preventing chronic disease [16,17]. Therefore, any suggestions on future sustainable diets for adolescents need to consider health-promoting aspects at the same time [18].

A frequently suggested approach to reduce the environmental impact from food is to avoid specific food categories such as meat (pescatarian diet), meat and fish (vegetarian diet), or any animal product (vegan diet), as these diets are associated with lower GHGE [19]. However, deficiencies in in the supply of some nutrients may affect the nutritional status of vegetarians and vegans negatively [20,21]. In 2019, the EAT-Lancet Commission suggested a healthy reference diet, based on studies of dietary patterns and health outcomes, that also had been evaluated against different environmental aspects [5]. The authors of the report called on all countries to make national adaptations to this generic diet. However, this diet neither has been fully controlled for nutritional adequacy, nor for specific cultural acceptability or affordability.

A comprehensive way to fulfil a broad range of criteria simultaneously is by optimization analysis through linear programming (LP) [22]. Using this methodology, diets that are nutritionally adequate, while at the same being reduced in GHGE and limited in cost, can be developed [22,23]. Additionally, this methodology has been shown to be successful for meeting cultural acceptability by minimizing the deviation from reported dietary patterns of the population [22,24,25,26].

The aim of the present study was to apply LP in designing nutritionally adequate and culturally acceptable diets with significantly reduced GHGE based on the current diet of adolescents in Sweden. We optimized the diet for four patterns, which varied based on their inclusion of animal foods (omnivores, pescatarians, vegetarians and vegan). The optimized diets were set to meet the maximum tolerable diet-related GHGE limit defined to keep the increase in global average temperatures below 1.5 °C above pre-industrial levels, as calculated by the World Wildlife Fund based on targets of the Intergovernmental Panel on Climate Change (IPCC) [27]. We also compared the optimized diets to the proposed EAT-Lancet diet [5].

## 2. Materials and Methods

### 2.1. Design and Dietary Data

This was a modeling study using linear programming to design nutritionally adequate and climate-friendly diets for omnivorous, pescatarian, vegetarian and vegan adolescents in Sweden. Dietary data were derived from the national dietary survey Riksmaten Adolescents 2016–2017, which is a school-based cross-sectional dietary survey of 3099 pupils from 130 schools including grades 5, 8 and 11 [28]. Consumed foods and their amounts were recorded using a validated, web-based 24-h recall method (RiksmatenFlex) on two non-consecutive days with the option to choose from 778 foods, of which 725 foods were recorded at least once [29]. The sample consisted of 55% girls and the participants were evenly distributed between the three grades: 34% pupils were between 10 and 11 years old in grade 5, 34% pupils were between 14 and 15 years in grade 8, and 32% pupils were between 17 and 18 years in grade 11. A more detailed description of the survey, methodology, data acquisition and evaluation can be found elsewhere [28].

### 2.2. Intake of Energy and Nutrients

Energy and nutrient intakes of the edible parts of foods as eaten (e.g., cooked rice) were automatically calculated through linkage with the Swedish Food Agency’s Food composition database version “Riksmaten Adolescents 2016–2017”. Added sugars are defined as all refined sugars added to foods during cooking and manufacturing, not including honey and unsweetened fruit juices (NNR 2012, EFSA) [30].

For optimization purposes, the reported intake of each food item (g/day) was standardized to 2410 kcal, i.e., the estimated energy requirement for a reference pupil/child as indicated in the Nordic Nutrition Recommendations 2012 [31]. The energy requirement was weighted according to the different age and sex groups in the study sample (see Table S1 and Section 2.7 for more details). The energy-proportional shares of each food for the reference pupil were calculated for modeling purposes and represented the pupils’ baseline food consumption. The reference energy intake 2410 kcal was also used as the pre-set daily energy constraint of all optimized diets.

### 2.3. Cost of Foods

The price of each food was searched for through the webpage “Matpriskollen” [32], which compares the prices of foods among twelve of Sweden’s largest food retailers. Based on the different available prices for a food item (including low budget, conventional and organic varieties), an average price was calculated for each food item.

### 2.4. Greenhouse Gas Emissions (GHGE) of Foods

The carbon dioxide equivalents (CO_2_eq) of foods were obtained from the Climate Database from Research Institutes of Sweden (RISE) [33], which is linked to the Swedish Food Agency’s Food composition database. It contains 2129 foods and reflects typical Swedish food supply/purchasing patterns. The Climate database builds on life cycle analyses [34,35], covering the GHGE of food production from resource extraction (cradle) to the factory gate. It contains values for carbon dioxide (CO_2_), methane (CH_4_), and nitrous oxide (N_2_O) that have been weighted in line with their respective global-warming potential over a 100-year period using factors recommended by the IPCC [36]. The combined emissions from the greenhouse gases from each food item yields a single value measured as kg of CO_2_eq per kg of food. We used the CO_2_eq-values which corresponded to the environmental impact of a food in its edible (e.g., boiled pasta) form.

### 2.5. Grouping of Foods

For analytical and descriptive purposes, foods were grouped into 22 food categories, based on the categorizations used in the RISE Climate Database (Bread; Cereals, other (including, e.g., pasta and rice); Nuts and seeds; Fruits and berries (including smoothies); Potatoes; Vegetables (e.g., tomatoes, cucumber, lettuce, bell pepper, carrots and a few vegetable-based dishes); Pulses (beans, lentils, peas and chickpeas); Meat substitutes; Dairy substitutes; Dairy, other (e.g., milk); Dairy, solid (including cheese, curd and yoghurt); Eggs; Pasta and rice dishes with meat/fish (e.g., composite dishes like pasta Bolognese); Poultry; Red/processed meat (e.g., beef, pork, including offal and meat-based dishes); Seafood (including fish, mussels and crabs); Oils; Fats, solid (e.g., butter, margarine); Drinks w/o milk; Sugar and sweets (including chocolate); Seasonings and sauces, and; Other (e.g., seeds, salt, sugar, jams).

**Figure 1:**
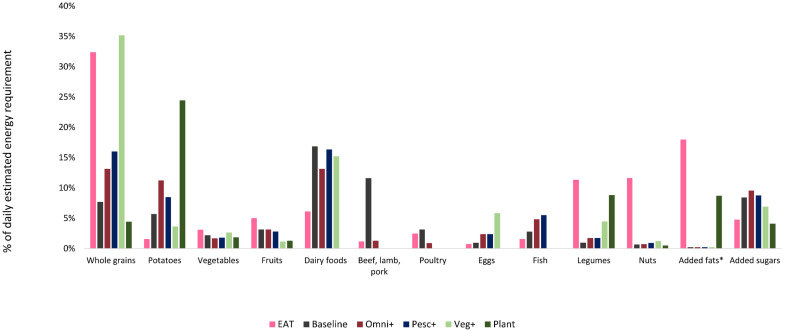
Comparison between the EAT-Lancet diet and (baseline and optimized) diets of Swedish Adolescents. Columns represent the percent of the daily estimated energy requirement for different food groups in the EAT-Lancet diet, the observed (baseline) diet, and in the four main optimized diets (“Omni+”, “Pesc+”, “Veg+”, ”Plant”). Food categories used in this comparison were based on the ones used for the EAT-Lancet diet [5]; * Added fats exclude dairy-based fats (such as butter), which are included in “Dairy foods”.

The baseline and optimized diets were also re-grouped in order to be comparable to the EAT-Lancet Commission’s food categorization used in the published report (Figure 1) [5]. This report applied the following categories: Whole grains (rice, wheat, corn and other); Tubers or starchy vegetables (e.g., potatoes); Vegetables; Fruits; Dairy foods (whole milk or equivalents, including butter); Beef, lamb and pork; Chicken and other poultry; Eggs; Fish; Legumes; Nuts; Added fats (unsaturated oils and saturated oils); and Added sugars.

### 2.6. Linear Optimization

Linear programming (LP) has successfully been applied to optimize goal determinants of diets while considering complex patterns of different constraints [22,37]. Briefly, LP is the application of an algorithm for maximizing or minimizing a given linear objective function (the variable to be optimized) subjected to a set of linear constraints (conditions to be met) on a list of decision variables (amount of each food item) [38]. A solution is found when all conditions can be met. If conditions are too strictly chosen, no solution is possible. Constraints that set the limit for the objective function’s ability of being minimized or maximized (e.g., those being met by exactly 100% with regards to its applied limit) are called “active constraints” [39]. Linear optimization was performed with the CBC (COIN-OR Branch and Cut) Solver algorithm, which is part of the Excel^®^ 2016 software add-in OpenSolver, V. 2.9.0 [40].

### 2.7. Nutritional Adequacy of Optimized Diets

Dietary reference values (DRVs) based on the Nordic Nutrition Recommendations 2012 [31], covering the nutritional needs of 97.5% of the population, were used as obligatory constraints for all solutions provided (Table S1). These constraints comprised the daily estimated energy requirements (EER), the recommended intake ranges for macronutrients, and the recommended intakes (RIs) for micronutrients [31]. The upper level for the salt intake was set to 6 g/day and the minimum value of fiber intake to 26 g/day [31]. In cases where the DRVs differed depending on age and/or sex, the nutritional constraints were weighted according to the DRVs and population size of the different age and sex groups in the study sample. All optimized diets met the DRVs for a reference pupil. Active nutrient constraints were identified for each solution (Table S2). As the bioavailability of iron is generally lower in vegetarian diets, an iron constraint of 1.8 times the RI provided by the Nordic Nutrition Guidelines was set for the “Veg“, the “Veg+” and the “Plant” models [41].

### 2.8. Total GHGE of the Baseline and Optimized Diet

The overall GHGE of the baseline food intake and the optimized diets was calculated as the sum of the products of the corresponding food weights and their specific CO_2_eq values as recorded in the Climate Database [33]. Based on the latest IPCC report [42], the World Wildlife Fund (WWF) has estimated that the GHGE from an individual’s diet should amount to a maximum of 11 kg CO_2_eq/week in order to keep global temperature increase below a 1.5 degrees, compared to preindustrial levels [27]. Hence, the GHGE upper limit for the daily diet was set to 1571 g CO_2_eq in all optimizations (see Section 2.11).

### 2.9. Total Cost of Baseline and Optimized Diet

From the total edible weight of each food item in the diets, the raw weight was calculated and multiplied by the specific cost to obtain the total cost of the baseline and the optimized diets, respectively.

### 2.10. Deviation from Baseline Diet

As the objective function of the LP model, we chose the minimization of the total relative deviation (TRD) from the baseline diet [26,43]. The minimized TRD from baseline was used as a proxy for cultural acceptability of the optimized diet solutions. The TRD is the (total) sum of the absolute (non-negative) values of the relative deviations (RDs) of the weight of a food in the optimized food supply from the reported intake of this food (Equation (1)).

(1) $${\mathrm{RD}}_{i}=\frac{M_{i}-m_{i}}{m_{i}}$$

In Equation (1), i indicates the running index of the food, M its mass in the optimized diet, and m the reported intake of that food. The TRD from all N food items in the model was calculated as the total sum of the absolute values of RDs:

(2) $$\mathrm{TRD}\;=\sum_{i=1}^{N}\mathrm{abs}\left({\mathrm{RD}}_{i}\right)$$

Since TRD is not a linear function and thus cannot be part of the linear equation system which LP builds upon, the non-negative values of RDi: RD1→RDN, with N being the number of foods included into the optimization, were created as described and applied previously [26,43]. In brief, the constraints applied to achieve the optimized absolute RD values were set so that the optimized values were greater or equal to both the actual negative and the positive RD value, which resulted in the optimized RD value being equal to the positive RD value, irrespective of whether the deviation was negative (reduced in comparison to the reported intake) or positive (increased). The decision variables were submitted to the following constraints (Formula (3)):

(3) $$\mathrm{abs}\left({\mathrm{RD}}_{i}\right)\;\geq \;(\mathrm{mi}-\mathrm{Mi})/\mathrm{mi}\;\mathrm{and}\;\mathrm{abs}\left({\mathrm{RD}}_{i}\right)\;\geq -(\mathrm{mi}-\mathrm{Mi})/\mathrm{mi}$$

Thus, for each standardized difference, its absolute (positive) value was selected because RDi, by definition, has to be greater than or equal to both the relative difference and its negative value.

To be able to control for unacceptably high amounts of individual food items in the optimized FBs, a maximum relative deviation of single foods from baseline was introduced, which had to be adapted during the optimization of each diet to reach a feasible solution (see also Section 2.11).

The average relative deviation (ARD) from the baseline food consumption was used as a proxy of similarity between the baseline and the optimized food consumption and was calculated by dividing the TRD by the total number of food items included in the model (N), as given in Formula (4):

(4) ARD=TRD/N

### 2.11. Models

The baseline food consumption was optimized following a strategy described previously [26]. For each of the dietary patterns, besides the vegan diet, the optimization was run without (Omni, Pesc, Veg, and Plant) and with (Omni+, Pesc+ and Veg+) the CO_2_eq constraint of 1.571 g per day [27]. Because the total CO_2_eq of the vegan diet (when modeled without a CO_2_eq constraint) was already below the WWF threshold, only a “Plant” diet was modeled. Hence, since the CO_2_eq constraint was not active in the vegan diet, a “Plant+” diet would have been identical to the “Plant” diet”. Hence, seven different LP models were applied (Table 1), which all had the minimization of the total relative deviation (TRD) from the baseline food consumption as the objective function. DRVs were implemented as obligatory constraints in all models (Table S1).

Model 1 (“Omni”) was run with nutritional constraints only, without constraining the GHGE (Table 1). In Model 2 (“Omni+”), the indicated CO_2_eq constraint was imposed. Consequently, Model 3 (“Pesc”, not CO_2_eq-constrained) and Model 4 (“Pesc+”, with CO_2_eq constraint), representing a pescatarian diet, were set up as per Models 1 and 2, but without red/processed meat and poultry meat products (=constrained to zero). Omitting specific food categories such as meat for the pescatarians increased other food groups to achieve isocaloric diets. This in turn required to increase the tolerated maximum relative deviations of single foods from baseline (right column in Table 1). In Models 5 and 6, representing an ovo-vegetarian diet (“Veg”, not CO_2_eq-constrained and “Veg+”, with CO_2_eq constraint, respectively) red/processed meat, poultry meat and seafood products were excluded. In the seventh model, representing a vegan diet (“Plant”), all animal products were made unavailable to the model. To avoid extreme deviations of single foods, the absolute RDs of individual food items were limited as much as possible until no feasible solution could be provided by the linear programming algorithm. This corresponded to +200% for Models 1–4, 600% for the vegetarian models 5 and 6, and 5000% for Model 7.

## 3. Results

The baseline GHGE based on the average food intake of an adolescent was 4.48 kg CO_2_eq/day (Table 2). This diet was lower than recommended in dietary fiber (90% coverage of DRV), polyunsaturated fatty acids (89% of DRV), vitamin D (83% of DRV), iron (89% of DRV), contained too much saturated fatty acids (135% of upper DRV) and sodium (157% of upper DRV) (Table S2).

In the four optimized diets, GHGE was reduced by 39–73% (Table 2). The lowest reduction in GHGE was achieved for omnivores (“Omni”, −39%) and the greatest reduction was observed in the vegan model (“Plant”, −73%) (Table 2). The ARD of the models ranged from 12.8% in the nutritionally adequate diet for omnivores (“Omni”) to 118% for the plant-based diet (“Plant”). Compared to baseline, the diet cost was reduced by approximately 20–30% in all optimized diets, with the pescatarian diet being the most affordable (approximately EUR 5/person/day) (Table 2).

Based on the exclusion of food groups when moving from an omnivorous to a plant-based diet, fewer foods were part of the modeled diets. For example, the “Omni+” model included the majority of the original foods (644 out of 725 foods), while the “Plant” diet contained only 313 foods (Table 2).

All optimized diets constrained to meet both nutritional and climate targets had a lower share of animal-based foods (Table 3, Figure 1). The “Omni+” diet contained 91% less Red/processed meat, 73% less Poultry, 65% less Pasta and rice dishes with meat/fish, and about half of the Solid dairy (mainly cheese) compared to the baseline diet (Table 3). However, considerable increases in other animal foods such as Eggs (+158%) and Seafood (+55%) were observed in the optimized “Omni+” diet (Table 3). In the pescatarian, vegetarian and vegan models, the categories Red/processed meat and Poultry were removed entirely (Figure 1). The “Pesc+” diet compensated for the absence of Red/processed meat and Poultry by increasing the share of Seafood (+72%) and Eggs (+158%).

The optimized diets also differed with respect to the amount and type of plant-based foods (Figure 1, Table 3). Pulses increased in all models, with the greatest changes seen in the “Plant” diet, where it increased more than ten-fold compared to baseline (Table 3). In contrast, the amount of Vegetables only increased in the vegetarian (“Veg” and “Veg+”) diet (Figure 1, Table 3). The amount of Potatoes increased in all optimized diets with the exception of the “Veg” and “Veg+” diets, the “Plant” diet showing the largest increase (+309%) (Table 3). Fruits remained almost unchanged (+9% in the “Omni” diet) or was reduced by up to 56% in the rest of the optimized diets (Figure 1, Table 3). Cereals such as pasta and oats increased in the models containing little or no animal products (“Veg”, “Veg+”, and “Plant”), and decreased in the optimized diets for omnivore and pescatarians (Table 3). Bread increased in all models with the exception of the “Plant” model.

The more the baseline dietary pattern was restricted, the more meat substitutes were included in the modeled diet (Table 3). For example, the “Omni+” solution contained the same amount of meat substitutes as the baseline diet, while the optimized “Plant” diet experienced a more than ten-fold increase in these foods compared to baseline. Overall, the LP algorithm was able to meet both nutritional and climate objectives without increasing the amount of Dairy substitutes with the exception of the “Plant” diet, where their amount increased by more than 50-fold, from roughly 9 g/day (baseline) to about 460 g/day (Table 3).

The active nutritional constraints of all models are shown in Table S2. Iron, selenium, and vitamin D were active lower-threshold constraints, while sodium was an upper-threshold active constraint in all models. Calcium was an active lower-threshold constraint in the models “Omni”, “Omni+” and “Pesc+”, but not in the “Pesc”, the “Veg” and the “Plant” models, which contained relatively high amounts of calcium-fortified dairy substitutes. Added sugars actively constrained the “Omni+” diet only. Achieving a minimum amount of polyunsaturated fatty acids was also an active constraint in the diets “Veg+”, “Pesc+”, and “Plant”. Vitamin A acted as an active constraint in all models except in the “Veg” diet.

When comparing the omnivorous EAT-Lancet diet [5] to our optimized models, pronounced differences were observed (Figure 1). Overall, the EAT-Lancet diet’s amounts were higher in Whole grain foods, Vegetables, Fruits, Legumes, Nuts, and Added fats, but lower in Potatoes, Dairy foods, Eggs, Fish and Added sugars than that provided by the optimized diets. The “Omni+” diet matched the EAT-Lancet diet with respect to red (beef, lamb, pork) meat. Naturally, the “Pesc+”, “Veg+” and” “Plant” diets did not match the suggested amounts of red or poultry meat in the EAT-Lancet diet. Similarly, the “Veg+” and “Plant” diets were below the maximum limit on Fish. The “Veg+” diet aligned to the EAT-Lancet diet in terms of “Whole grains”, whereas the “Plant” diet was the only diet mirroring the target for “Added sugars”. The average relative deviation for all food groups between the EAT diet and the optimized diets (i.e., the sum of absolute relative deviations divided by the number of food groups compared) was 134, 136, 127, and 181 percent for the “Omni+”, “Pesc+”, “Veg+” and “Plant” diets, respectively (Figure 1).

## 4. Discussion

In this study, we demonstrated that nutritionally adequate diets, which align with the maximum tolerable diet-related GHGE limit defined to keep the increase in global average temperatures below 1.5 °C above pre-industrial levels, can be achieved for four different dietary patterns. Simply modifying the current diet of Swedish adolescents to meet DRV values resulted in a 39% decrease in GHGE, which was mainly achieved by a pronounced reduction in solid dairy foods (cheese and curd) and meat. Relative to the baseline diet, the GHGE in the nutritionally adequate pescatarian model (“Pesc”) was reduced by 59%, by 62% in the vegetarian model (“Veg”) and by 73% in the vegan (“Plant”) model. The amount of CO_2_eq in the baseline diet of the adolescents was 4.5 kg/day, a value that is comparable to the ~5 kg CO_2_eq/day previously reported for adults [15]. This means that in order to reach the threshold of 1.57 kg CO_2_eq/day proposed by the WWF [27], the GHGE had to be reduced by 65% [26,43]. Only the optimized, nutritionally adequate vegan diet (“Plant”) dropped below the IPCC/WWF threshold without further active restriction of the model’s GHGE. The exclusion of food groups in the pescatarian, vegetarian and vegan diets along with constraining the GHGE increased the deviation from the baseline diet, especially for the optimized vegetarian and vegan models as compared to the omnivoric or pescatarian solutions. The optimized diets, despite being nutritionally adequate and reaching the recommended GHGE level, did not align very well with the food-group pattern of the EAT-Lancet diet [5].

Constraining the reported food intake to meet the DRVs alone resulted in a marked reduction of GHGE, which is in line with previous findings [26,44]. However, the 39% reduction in GHGE achieved in the “Omni” diet is surprisingly high compared to previous studies in UK adults where the reduction was 17% [44]. This can be explained mainly by the DRV-enforced reduction of saturated fatty acids and sodium as well as the increased inclusion of foods that are rich in fiber and polyunsaturated fatty acids. These changes increase the share of plant-based foods with a low climate impact at the expense of animal-based foods, the consumption of which is comparably high in this sub-population [16].

The climate-friendly and nutritionally adequate food profile for omnivores (“Omni+”), which mimics the dietary habits of Swedish adolescents the best, showed a more pronounced trend towards reduction of meat, poultry, and solid dairy than the non-GHGE-constrained alternative (“Omni”). This reduction was compensated by an increase in the amounts of less GHGE-intense animal products such as eggs, but a major part of the substitution was based on an increased inclusion of pulses, potatoes, and bread. Table 4 summarizes the optimized solution of the “Omni+” diet. Others have also calculated climate-friendly diets for the general population [5,45], but without ensuring nutritional adequacy.

In the pescatarian model (“Pesc+”), the optimized solution is very similar to that of the omnivore diet (“Omni+”), except that meat and meat products are replaced by moderately increased amounts of fish, meat substitutes, and dairy products (Table S3). Both the omnivorous and the pescatarian diets include increased amounts of fish compared to the baseline diet. Presently, a large part of the fish consumed originates from marine capture fisheries [46], which explains the low CO_2_eq-value of this micronutrient- and protein-rich commodity. However, 96 of the world’s fish stocks are either moderately or fully exploited, or over-fished [47]. Farmed fish such as salmon has GHGE values comparable to or even higher than that of poultry, pork and dairy and can in addition be a source of eutrophication [48]. If a high proportion of the population follows the recommendation to increase the intake of farmed or captured fish, the biodiversity of certain fish types should be considered in addition to their production-related climate impacts.

The climate-friendly solution for vegetarians includes considerably increased amounts of dairy and meat substitutes (which are mostly mycoprotein-, pea- or soy-based products), pulses, bread, potatoes, and some vegetables to compensate for excluding meat and fish (Table S4). Vegetarian diets have been recommended as a principal approach to reduce the climate impact of the diet, though again, these are not based on calculations that ensure full nutritional adequacy [49,50,51] and may increase the risk of micronutrient deficiencies. For example, one third of Swedish female adolescents have low iron stores [17]. Excluding meat and fish from the diet may result in lower iron intakes as well as in a diet with a lower iron bioavailability. Haem iron, found in meat, is more readily absorbed than non-haem varieties. Furthermore, meat and fish enhances absorption of iron from plant-based foods [31]. Absence of haem iron in the diet may affect iron status negatively in vulnerable populations and highlights the need for reliable guidance on what to replace meat with and how to combine foods to increase bioavailability [52]. Therefore, in the optimized diets building on the Veg, Veg+, and Plant models, a higher minimum threshold of iron was set as recommended by the US Institute of Medicine [41]. The high bioavailability of ferritin-bound iron in legumes may also help to overcome this shortcoming [53].

Excluding all animal products in the ”Plant” model resulted in a considerable inclusion of (mostly fortified) meat and dairy substitutes along with an increased intake of pulses, potatoes and non-dairy fats (Table S5). Although plant-based foods are considered to have a low bioavailability of iron, calcium, vitamin D and B12 and although the minimum threshold was raised for iron, all applied DRV values were covered by the optimized solution for vegans. Besides iron, a sufficient supply of calcium and vitamin B12 was also guaranteed even for the vegetarians and vegans. This was primarily achieved due to the high fortification of dairy replacements with these micronutrients. These results mirror a recent optimization study on Dutch eating habits, where the optimized diet for vegans met DRVs for vitamin B12 and calcium only through the inclusion of sufficiently high amounts of fortified soy milk [54]. This raises the question as to whether fortification or, alternatively, supplementation are acceptable ways forward to reduce diet-related GHGE. More studies on replacement food, fortification, and health outcomes are clearly needed. Furthermore, the production of meat and dairy replacements raises concerns about other environmental indicators. For example, plant-based milk replacements may contribute to water scarcity, deforestation and biodiversity loss [55], although this may vary depending on type of product and country. Further investigations are needed to fully understand how the “Veg+”- and “Plant” diets would impact the full range of health and environmental indicators in the context under study.

As is evident from Figure 1, the optimized “Plant” diet contained the lowest amount of whole grains and the highest amount of potatoes. Furthermore, the amount of vegetables (excluding legumes), fruits and nuts was comparably low. This food pattern differs somewhat from other recommendations on plant-based diets. For example, recent recommendations on plant-based diets for adolescents [56] emphasize the inclusion of whole grains, legumes, nuts and seeds, vegetables, and fruits to the diet. These differences are likely to result from the fact that environmental aspects have so far insufficiently been considered in the development of food-based recommendations. Studies show that the increased inclusion of fruit and vegetables in the diet, although beneficial from a health point of view, can lead to higher environmental impacts [6,7,8,57], or be less effective in reducing them [58]. Furthermore, diets optimized to meet nutritional constraints only [59,60] have been shown to have higher climate footprints. On the other hand, self-selected, plant-based diets with lower climate footprints have been shown to lead to the overconsumption of refined sugars [14,15]. This stands in contrast to the optimized “Plant” model, that had the lowest amounts of added sugars. In summary, these findings add to the challenges in defining the sustainability of diets. It is, therefore, advisable to use a holistic approach such as linear programming (that consider both health and environmental priorities) in the definition of food-based recommendations for different dietary patterns.

Our findings reveal that neither the baseline nor the optimized diets of Swedish adolescents align with the EAT-Lancet Commission’s dietary recommendation for a sustainable diet [5]. This could be due to three reasons: (1) we optimized for similarity to the reported food consumption patterns of Swedish adolescents to achieve a high cultural acceptability instead of using the EAT-Lancet diet as the reference; (2) our models were all constrained to ensure the fulfilment of 27 DRVs, which the EAT-Lancet diet was not; (3) the EAT-Lancet diet considered additional dimensions of sustainability such as blue water footprint, land use change and animal welfare, which were not considered in the study at hand. In contrast to the EAT-Lancet diet, the models “Omni” and “Omni+” include significant amounts of dairy, fish, and eggs. Another difference is the much higher amount of potatoes and a markedly lower amount of legumes in the optimized diets as compared to the EAT-Lancet diet. Potatoes, commonly consumed in the Swedish adolescent population, are a dominant and nutritious staple-crop in Sweden considered to be healthy [61]. Although all optimized diets diverged from the EAT diet, the Veg+ diet was the most similar on a food group level.

Despite the discrepancies, some similarities between the EAT diet and the optimized diets can be found. For example, the optimized vegetarian diet (“Veg+”) matched it with respect to Whole grains and Vegetables and the optimized vegan (“Plant”) diet was comparable in terms of Legumes and Added sugars. Furthermore, like the EAT-Lancet diet, both Omni models suggest a comparable amount of red meat and poultry to achieve a nutritious and climate-friendly diet. In contrast to the EAT-Lancet diet [5], our diets optimized for similarity may be easier to achieve for adolescents in the Swedish population.

Food-based dietary guidelines (FBDGs) were not considered as constraints in the optimizations. Today, the Nordic Nutrition Recommendations has quantifiable FBDG regarding fruit and vegetables (500 g/day) and fish (2–3 times per week) [62]. Only the “Plant” model met the Swedish FBDGs’ recommended intake of 500 g fruits and vegetables (including pulses) per day. The LP algorithm in general did not favor either fruits or vegetables which can be explained by the fact that fruits and certain types of vegetables (such as tomatoes, cucumbers, and onions) may provide smaller amounts of nutrients per gram of CO_2_eq compared to other foods such as starchy vegetables and pulses. It thus mirrors research showing that a generous inclusion of fruit and vegetables into the diet can result in higher dietary environmental impacts [6,7,8]. Another plausible explanation is that our solutions were optimized to be as similar as possible to the baseline diet, where the intake of fruit and vegetables was relatively low. This finding aligns well with findings from the Netherlands, Denmark, and Estonia, where nutritionally adequate diets optimized for acceptability did not meet national FBDG-targets for fruit and vegetables [37,54,63].

One strength of our research is that it highlights the potential of optimized diets, such as those achieved in this study, to be translated into sustainable food-based dietary guidelines. However, for this to happen, other scientific evidence such as the impact on additional environmental factors (blue water usage, land use change, and biodiversity) and other legitimate factors (food safety) must also be considered. Furthermore, additional detailed information may be necessary to be included such as the prioritization of local vs. imported products. Further adaptation towards individual needs may also be necessary before formulating food-based dietary guidelines with support from linear optimization.

Future modeling studies should investigate the feasibility and need for including both DRVs and FBDG in the models as well as aspects on food safety and other environmental aspects such as biodiversity, pollutants, blue water use.

The GHGE values indicated include only the CO_2_eq to the factory gate, but not the GHGE associated with transportation to the retailer and to the home or food preparation. Therefore, the final CO_2_eq values from different foods might be slightly higher than those calculated in this study.

As the data were recorded in 2016–2017, dietary habits might have changed moderately since then. Furthermore, all optimized diets cover the estimated micronutrient intake of 97.5% of the population. This may be unnecessarily high when using the suggested diets to fulfill average intakes for population groups but guarantees on the other hand the applicability of the optimized diets also for individuals. Another limitation was that no new foods were introduced into the models. There are many new meat and dairy substitutes emerging on the market [64,65]. Including these foods in the optimization of diets could provide certain benefits for the environment without compromising nutritional adequacy [66]. Future studies should further explore the health impacts and environmental effects of also including such foods in the modeling. Since the dietary survey data was averaged, data on the food intake of pescatarians, vegetarians and vegans were not available during optimization. Therefore, the optimization may also be limited for the groups of pescatarians, vegetarians and vegans, as the reported omnivore diet was used as reference. In the case of optimized non-omnivoric diets, the RD represents the deviation after changing to a pescatarian, vegetarian or vegan diet. It is not representative of individuals who already practice these diets.

One of the strengths of this study is that it provides the first guidance for achieving more climate-friendly diets based on the dominating omnivoric dietary pattern of adolescents in Sweden. The results feed into the discussion on how future FBDGs should be shaped. Since comprehensive fiscal measures such as taxes and subsidies to influence on people’s food choices are currently not promoted by decision makers in Sweden, information and nudging may be the obvious policy tool available to affect consumer behavior [67]. Therefore, it is critical that messages are simple and clear, yet still sufficiently informative to avoid unintended substitutions and adverse outcomes [18].

## 5. Conclusions

The results of this study show that an affordable, nutritionally adequate diet with a considerably reduced GHGE can be achieved for omnivorous, pescatarian, vegetarian and vegan Swedish adolescents. Particularly for vegetarians and vegans, this means large deviations from the current reported food pattern. However, even in the climate-friendly diet for omnivores, a considerable reduction in the consumption of red/processed meat (pork and beef), poultry, and solid dairy (cheese) along with an increased intake of potatoes and fish would be needed to meet the desired climate targets. Excluding meat and fish from the diet demands the inclusion of substitutes for meat and dairy, which are fortified with calcium and the vitamins D and B12 to ensure nutritional adequacy. Food fortification is an issue that needs to be discussed in future diet modifications. Our findings can contribute to national recommendations that are simple and clear, yet still sufficiently informative to avoid unintended and adverse outcomes for both human and planetary health. The optimized omnivorous, nutritionally adequate diet in this study differed in several aspects from the EAT-Lancet diet, indicating that there are several ways to define sustainable diets but also that the cultural dietary context is likely to play an important role in characterizing such diets for specific populations. This study provides a basis that can be used in the development of food-based dietary guidelines on affordable, nutritionally adequate diets that are low in GHGE. This methodology can also be applied for other age groups and countries after the basis of the optimization has been adapted to the specific geographical and cultural dietary context.

## Figures and Tables

**Table 1:** Names and characteristics of all applied models.

Model Number and Acronym	Objective Function (Minimum)	Foods Available	Nutritional Constraints	CO_2_eq Constraint	Acceptability Constraint Max RD ^b^ for Food Items
1. Omni	TRD ^a^ from baseline diet	All food items	Meet all DRVs ^c^	Not applied	+200%
2. Omni+				Max. 1571 g CO_2_eq	+200%
3. Pesc		No meat or poultry products		Not applied	+200%
4. Pesc+				Max. 1571 g CO_2_eq	+200%
5. Veg		No red/processed meat, poultry meat, or seafood products	Meet all DRVs, minimum iron intake constraint 1.8 × the DRV of omnivores	Not applied	+600%
6. Veg+				Max. 1571 g CO_2_eq	+600%
7. Plant		No animal products		Not applied ^d^	+5000%

^a^ Total relative deviation. ^b^ Relative deviation from baseline food consumption. ^c^ Estimated energy requirements (EERs), recommended intake ranges for macronutrients, recommended intakes (RIs) for micronutrients [31]. ^d^ A CO_2_eq constraint was not needed since the “Plant” model (without a CO_2_eq constraint) resulted in a total CO_2_eq below 1571 g CO_2_eq.

**Table 2:** Cost, average relative deviation (ARD), min/max relative deviation (RD) values, CO_2_eq values, and the number of foods removed, reduced or increased in the optimized diets for omnivores, pescatarians, vegetarians, and vegans compared with the baseline consumption of Swedish adolescents.

Diet ^a^	CO_2_eq Constraint (g/Day)	Max RD Set (%)	CO_2_eq (g/Day)	ARD (%)	Cost (SEK ^b^)	FB Weight	of Foods Available ^c^	of Foods Unavailable ^c^	of Foods Removed by Optimization	of Foods Removed in Total	of Foods with Reduced Amount	of Foods with Increased Amount
Baseline	none	-	4481	0.0	77.24	2130	725	0	0	0	0	0
Omni	none	200	2729	12.8	60.71	2018	725	0	47	47	319	359
Omni+	1571	200	1571	21.1	61.73	1843	725	0	81	81	300	344
Pesc	none	200	1861	29.4	53.14	2144	596	129	13	142	265	314
Pesc+	1571	200	1571	31.4	51.43	1925	596	129	17	146	272	306
Veg	none	600	1682	72.0	61.07	1916	550	175	74	249	214	262
Veg+	1571	600	1571	73.0	59.14	1793	550	175	77	252	209	264
Plant	none	5000	1227	118	57.28	2034	334	391	21	412	145	168

^a^ All optimized diets meet the dietary recommended values (DRVs). ^b^ Swedish Krona (1 SEK = approximately 0.10 Euro). ^c^ Availability based on type of diet (e.g., all red meat was made unavailable in the “Pesc” and “Pesc+” models).

**Table 3:** Baseline intakes of food groups among Swedish adolescents and relative changes in food groups after optimization of different dietary models.

Model Name	Baseline Diet (g/Day)	Omni (% Change)	Omni+ (% Change)	Pesc (% Change)	Pesc+ (% Change)	Veg (% Change)	Veg+ (% Change)	Plant (% Change)
Model		1	2	3	4	5	6	7
CO_2_eq limit (1571 g) applied		no	yes	no	yes	no	yes	no
Bread	85.7	89.1	102	94.5	156	160	160	−59.3
Cereals. other	218	−30.4	−26.5	7.0	−15.0	15.7	20.2	41.2
Nuts and seeds	4.2	16.7	3.0	16.7	24.3	55.0	55.0	−24.4
Fruits and berries	121	9.3	0.0	−21.2	−6.7	−44.8	−56.3	−55.1
Potatoes	121	19.1	97.4	7.1	33.6	−14.5	−36.7	309
Vegetables	104	−2.6	−13.0	−6.9	−6.3	188	121	−3.3
Pulses	21.7	37.4	82.4	82.3	82.3	348	348	1125
Meat substitutes	5.7	0.0	0.0	121	40.4	440	439	1165
Dairy substitutes	9.2	0.0	0.0	0.0	0.0	0.0	0.0	4867
Dairy, other	490	−6.1	−19.8	51.3	2.3	12.1	1.6	−100
Dairy, solid	25.7	−52.0	−54.8	−52.0	−52.0	−80.0	−88.8	−100
Eggs	13.0	132	158	139	158	533	533	−100
Pasta and rice dishes with meat/fish	111	−73.5	−65.9	−99.0	−99.0	−100	−100	−100
Poultry	44.2	−10.3	−73.1	−100	−100	−100	−100	−100
Red/processed meat	161	−63.5	−90.8	−100	−100	−100	−100	−100
Seafood	44.8	32.2	55.0	62.5	71.9	−100	−100	−100
Oils	0.1	0.0	0.0	0.0	0.0	0.0	0.0	−21.6
Fats. solid	10.7	45.2	51.4	15.4	73.3	82.3	83.4	215
Drinks w/o milk	425	10.9	−27.0	−13.4	−18.7	−73.0	−69.8	−58.1
Sugar and sweets	35.7	49.1	34.9	49.2	24.1	−20.2	15.2	−53.6
Seasonings and sauces	79.1	−40.0	−30.8	−19.2	−33.1	−66.2	−60.3	−80.6
Other	0.2	0.0	0.0	0.0	174	521	521	−100

**Table 4:** Quantities of food groups for an omnivorous diet with 2410 kcal, generating a maximum of 1571 g of CO_2_eq/day, based on the “Omni+” model.

About 180 g of (whole grain) bread and approximately 160 g of other cereals (rice, pasta, etc.) per day
At least 40 g of pulses per day At least 230 g of potatoes per day
Around 220 g of fruits and vegetables per day
About one egg per day
One portion of fish and other seafood (~150 g) every second day, every third portion being oily fish
Around one portion (~190 g) of meat, meat dishes and poultry per week (preferably pork, poultry, and offal such as liver and blood products rather than beef)
Not more than 400 g of dairy products and about one slice of cheese (15 g) per day
A handful of nuts and seeds per week (~30 g)

## Data Availability

Data used for these analyses can be made available from the Corresponding author upon reasonable request.

## Supplementary Materials

The following are available online at https://www.mdpi.com/article/10.3390/nu13082507/s1, Table S1: Dietary reference values applied as constraints in the linear programming models, Table S2: Nutrient coverage for baseline intake and optimized solutions, Table S3: Quantities of food groups for a pescatarian diet generating a maximum of 1571 g of CO_2_eq per day, based on the “Pesc+” model, Table S4: Quantities of food groups for a pescatarian diet generating a maximum of 1571 g of CO_2_eq per day, based on the “Veg+” model, Table S5: Quantities of food groups for a pescatarian diet generating a maximum of 1571 g of CO_2_eq per day, based on the “Plant” model.
