# Supplementary material for: Designing Nutritionally Adequate and Climate-Friendly Diets for Omnivorous, Pescatarian, Vegetarian and Vegan Adolescents in Sweden Using Linear Optimization
Source: Nutrients. 2021 Jul 22;13(8):2507. doi: 10.3390/nu13082507 (PMC8398609; doi:10.3390/nu13082507)
Supplement: Supplementary file 1 [file nutrients-13-02507-s001.zip › Table S1.pdf]

Table S1. Dietary reference values applied as constraints in the linear programming models.

	Unit	Lower limit	Upper limit
Energy[a,b]	kcal	2410	
Carbohydrates[a]	g	271	361.0
Fat[a]	g	66.9	107.0
Protein[a]	g	60.3	120.5
Fiber[a]	g	26.0	
SFA[a]	g		26.8
MUFA[a]	g	26.8	53.6
PUFA[a]	g	13.4	26.8
Vitamin A[b]	µg	726	
Vitamin E[b]	mg	8.4	
Tiamine[b]	mg	1.2	
Riboflavine[b]	mg	1.4	
Vitamin C[b]	mg	66.7	
Niacin[b]	mg	16.2	
B6[b]	mg	1.3	
B12[a]	µg	2.0	
Folate[b]	µg	285	
Phosphor[a]	mg	668	
Iodine	µg	150	
Iron[b]	mg	12.2	
Calcium[a]	mg	868	
Potassium[b]	mg	3.2	
Magnesium[b]	mg	301	
Sodium	mg		6.0
Selenium[b]	µg	49.6	
Zink[b]	mg	9.2	
Added sugars	g		60.3

[a]Adjusted for age differences, with 34% of pupils in grade 5, 34% in grade 8 and 32% in grade 11.
[b]Adjusted for age and sex differences with 55% females and 45% males.
