# Supplementary material for: Designing Nutritionally Adequate and Climate-Friendly Diets for Omnivorous, Pescatarian, Vegetarian and Vegan Adolescents in Sweden Using Linear Optimization
Source: Nutrients. 2021 Jul 22;13(8):2507. doi: 10.3390/nu13082507 (PMC8398609; doi:10.3390/nu13082507)
Supplement: Supplementary file 1 [file nutrients-13-02507-s001.zip › Table S3.pdf]

Table S3. Quantities of food groups for a pescatarian diet generating a maximum of 1571 grams of CO_2eq per day, based on the "Pesc+"-model.

- About 200 g of (whole grain) bread and up to 200 g of other cereals (rice, pasta, etc.) per day
- At least 40 g of pulses per day
- At least 160 g potatoes per day
- Around 220 g of fruits and vegetables per day
- About one egg per day
- One portion of fish and other seafood (~150 g) every second day, every third portion being oily fish
- Small amounts of meat- and dairy substitutes in your diet (about one portion every week of each)
- Not more than 500 g of dairy products and about one slice of cheese (15 g) per day
- A handful of nuts & seeds per week (~40 g)
