# Supplementary material for: Designing Nutritionally Adequate and Climate-Friendly Diets for Omnivorous, Pescatarian, Vegetarian and Vegan Adolescents in Sweden Using Linear Optimization
Source: Nutrients. 2021 Jul 22;13(8):2507. doi: 10.3390/nu13082507 (PMC8398609; doi:10.3390/nu13082507)
Supplement: Supplementary file 1 [file nutrients-13-02507-s001.zip › Table S4.pdf]

Table S4. Quantities of food groups for the optimized vegetarian diet generating a maximum of 1571 grams of CO_2eq, based on the "Veg+"-model.

- About 220 g of (whole grain) bread and up to 270 g of other cereals (rice, pasta, etc.) per day
- At least 100 g of pulses per day
- At least 80 g potatoes per day
- Approximately 230 g of vegetables per day around one fruit (~60 g) per day
- About two eggs per day
- Two small portions of meat substitutes (220 g) and one small portion of (fortified) dairy substitutes (~100 g)
- Not more than 500 g of dairy products per day and about two slices of cheese (15 g) per week
- A large handful of nuts & seeds per week (~50 g)
