# Supplementary material for: Designing Nutritionally Adequate and Climate-Friendly Diets for Omnivorous, Pescatarian, Vegetarian and Vegan Adolescents in Sweden Using Linear Optimization
Source: Nutrients. 2021 Jul 22;13(8):2507. doi: 10.3390/nu13082507 (PMC8398609; doi:10.3390/nu13082507)
Supplement: Supplementary file 1 [file nutrients-13-02507-s001.zip › Table S5.pdf]

**Table S5.** Quantities of food groups for the optimized vegan diet generating about 1227 grams of $CO_2$eq, based on the “Plant”-model.

- Not more than around one slice (<40 g) of bread (whole grain) per day
- Up to 300 g of other cereals (rice, pasta, etc.) daily
- At least 260 g of pulses per day
- At least 500 g potatoes per day
- One fruit (~60 g) and approximately 100 g of vegetables per day
- One portion of meat replacements (~150 g) every second day and large amounts of (fortified) dairy substitutes (~460 g) daily
- A handful of nuts & seeds (~30 g) per week
- About 35 g of (fortified) fats of plant origin per day
