# Supplementary material for: Designing Nutritionally Adequate and Climate-Friendly Diets for Omnivorous, Pescatarian, Vegetarian and Vegan Adolescents in Sweden Using Linear Optimization
Source: Nutrients. 2021 Jul 22;13(8):2507. doi: 10.3390/nu13082507 (PMC8398609; doi:10.3390/nu13082507)
Supplement: Supplementary file 1 [file nutrients-13-02507-s001.zip › Table S2.pdf]

Table S2. Nutrient coverage for baseline intake and optimized solutions. Unless indicated otherwise, all nutrient values represent the relative coverage (%) of the recommended values.

	**Unit**	**Baseline[a]**	**Omni[b]**	**Omni+[b]**	**Pesc+[b]**	**Veg+[b]**	**Plant[b]**
CO_{2eq}. average per day	g	4481	2729	1571	1571	1571	1211
	change (%)	na	-39	-65	-65	-65	-73
Cost average per day	SEK	77.2	60.71	61.73	51.43	59.40	57.28
	change (%)	na	-21	-20	-33	-23	-26
ARD	(%)	na	12.8	21.1	31.4	73.0	118.0
Carbohydrates	Lower	104	118	120	122	117	132
	Upper	78	88	90	91	88	99
Fat	Lower	135	120	120	116	114	101
	Upper	85	75	75	72	72	63
Protein	Lower	171	143	130	128	137	105
Fiber	Lower	**90**	107	126	133	212	202
Saturated fatty acids	Upper	**135**	**100**	**100**	98	96	59
Monounsaturated fatty acids	Lower	131	121	120	112	107	**100**
Poly unsaturated fatty acids	Lower	**89**	**100**	**100**	**100**	**100**	132
Vitamin A	RI	108	101	**100**	**100**	111	**100**
Vitamin D	RI	**83**	**100**	**100**	**100**	**100**	**100**
Vitamin E	RI	154	176	183	177	200	258
Thiamine	RI	137	145	144	150	198	366
Riboflavin	RI	145	133	133	142	190	314
Vitamin C	RI	149	186	150	130	125	324
Niacin	RI	148	147	132	119	118	256
Vitamin B6	RI	179	190	187	177	187	447
Vitamin B12	RI	287	236	234	213	178	127
Folate	RI	119	125	124	129	199	291
Phosphor	RI	265	236	232	230	283	229
Iodine	RI	153	**100**	114	106	106	107
Iron	RI	**89**	**100**	**100**	**100**	**100**	**100**
Calcium	RI	134	**100**	**100**	**100**	116	104
Potassium	RI	104	107	109	103	117	160
Magnesium	RI	114	120	129	131	199	153
Sodium	Upper	**157**	**100**	**100**	**100**	**100**	**100**
Selenium	RI	112	**100**	**100**	**100**	**100**	**100**
Zink	RI	146	117	105	106	156	103
Added sugars	Upper	84	**100**	94	86	68	40

[a]Numbers in bold highlight the nutrients which did not meet the dietary reference values in the baseline diet.
[b]Numbers in bold are the model's active constraints in the optimized diets.
ARD = Average Relative Deviation
